# A novel gastric defect closure method using lines and reopenable clips with the through-the-scope tying technique

**DOI:** 10.1055/a-2356-7640

**Published:** 2024-07-15

**Authors:** Junki Toyoda, Tatsuma Nomura, Takanobu Mitani, Yuto Ikadai, Sase Tomohiro, Tomonori Saito, Katsumi Mukai

**Affiliations:** 1 Gastroenterology, Suzuka General Hospital, Suzuka, Japan; 2 37071 Gastroenterology, Ise Red Cross Hospital, Ise, Japan

Gastric endoscopic submucosal dissection (ESD) defect closure is a difficult procedure in terms of achieving complete closure. Recently, a useful method for closing defects using a line and needle was reported 1 ; however, the endoscope must be withdrawn for ligation of the lines. Therefore, we have devised a new closure method, the “through-the-scope tying technique” (TTST), in which the lines are tied by hand outside of the body and can be ligated through the endoscope accessory channel.

A 68-year-old man presented with a 40-mm post-ESD defect in the gastric antrum. We performed defect closure using the TTST ( Fig. 1 ; Video 1 ). The TTST is a closure technique using two lines (0.16-mm polyethylene line) and reopenable clips (SureClip; MicroTech, Nanjing, China). First, a reopenable clip with two attached lines, one tied to each tooth, was placed via the accessory channel into the central part of the muscle layer of the post-ESD defect. Another reopenable clip, with one of the lines passed through the hole in one tooth, was inserted and placed on the defect edge using the reopenable clip over-the-line method (ROLM) 2 3 . Similarly, a third reopenable clip with the other line passed through the hole in the tooth was placed at the contralateral edge of the defect. The two lines were then tied twice by hand outside the body, in a double knot ( Video 1 ). The ends of the two lines were then passed through the tooth holes on either side of a clip and the knot was fed through the accessory channel with the reopenable clip closed. Finally, another knot was tied outside the body and delivered with the clip in the same way to leave the lines securely tied. The lines were cut using the locking clip technique 4 .

**Fig. 1:**
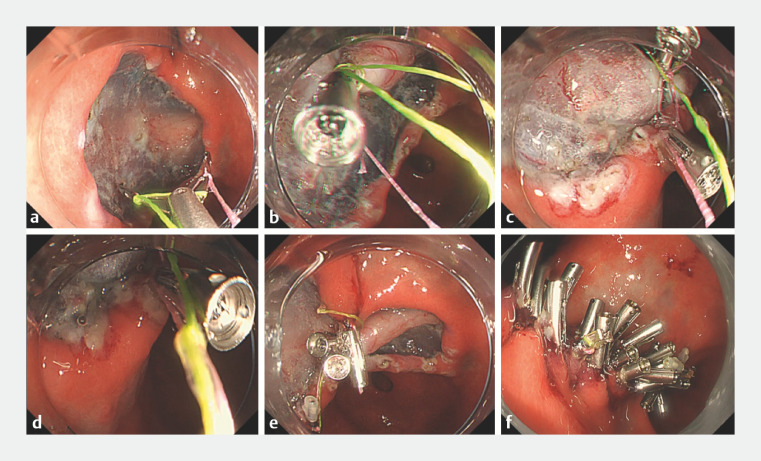
Endoscopic images of the new mucosal defect closure method using the “through-the-scope tying technique” (TTST) and reopenable clip over-the-line method (ROLM) showing: **a, b** a clip with two attached lines being placed in the central muscle layer of a 40-mm mucosal defect; **c** a clip with one of the lines passing through one of its teeth placed at the defect edge; **d** a knot, created in the two lines by tying them together by hand outside of the body, that ligates the clips after being pushed through the accessory channel with a closed reopenable clip acting as a knot pusher device; **e** the mucosal defect after TTST has been performed; **f** complete closure of the mucosal defect with additional ROLM.

Mucosal defect closure method using the through-the-scope tying technique (TTST) and ROLM for a large 40-mm gastric post-ESD defect.Video 1

With this technique, the defect could be completely closed with additional ROLM, as the muscle layer and mucosal layer had been firmly fixed by the TTST. The TTST is a novel method of tying a knot using a line and delivering the knot through the accessory channel, without having to withdraw the endoscope.

Endoscopy_UCTN_Code_TTT_1AO_2AO

## Notes

Endoscopy E-Videos https://eref.thieme.de/e-videos
*E-Videos* is an open access online section of the journal *Endoscopy* , reporting on interesting cases and new techniques in gastroenterological endoscopy. All papers include a high-quality video and are published with a Creative Commons CC-BY license. Endoscopy E-Videos qualify for HINARI discounts and waivers and eligibility is automatically checked during the submission process. We grant 100% waivers to articles whose corresponding authors are based in Group A countries and 50% waivers to those who are based in Group B countries as classified by Research4Life (see: https://www.research4life.org/access/eligibility/ ). This section has its own submission website at https://mc.manuscriptcentral.com/e-videos .
